# Study of high-altitude cerebral edema using multimodal imaging

**DOI:** 10.3389/fneur.2022.1041280

**Published:** 2023-01-26

**Authors:** Changyou Long, Haihua Bao

**Affiliations:** Department of Medical Imaging Center, Qinghai University Affiliated Hospital, Xining, China

**Keywords:** high altitude, cerebral edema, classification, computed tomography, magnetic resonance imaging

## Abstract

**Objective:**

To analyze the brain imaging features of high-altitude cerebral edema (HACE) using computed tomography (CT) and multi-sequence magnetic resonance imaging (MRI) and to explore its injury characteristics.

**Materials and methods:**

We selected 30 patients with HACE diagnosed between January 2012 to August 2022 as the experimental group and 60 patients with dizziness on traveling from the plain to the plateau or from lower altitude to higher altitude in a short period of time as the control group. We collected general clinical data from the experimental group and classified it according to clinical symptoms. In both groups, we then performed a head CT and multi-sequence MRI (T1WI, T2WI, FLAIR, and DWI). Among them, nine patients with HACE were also scanned using susceptibility-weighted imaging (SWI). Finally, we analyzed the images.

**Results:**

According to clinical symptoms, we divided the 30 cases of HACE into 12 mild cases and 18 severe cases. There was no significant difference in sex, age, leukocyte, neutrophil, or glucose content between mild and severe HACE. The sensitivity and specificity of the MRI diagnosis were 100 and 100%, respectively, while the sensitivity and specificity of the CT diagnosis were 23.3 and 100%, respectively. The distribution range of deep and juxtacortical white matter edema was significantly larger in severe HACE than in mild HACE (*p* < 0.001). The corpus callosum edema distribution range in severe HACE was significantly larger than that in mild HACE (*p* = 0.001). The ADC value of the splenium of the corpus callosum was significantly lower in severe HACE than in mild HACE (*p* = 0.049). In mild and severe HACE, the signal intensity of the DWI sequence was significantly higher than that of conventional MRI sequences (T1WI, T2WI, FLAIR) (*p* = 0.008, *p* = 0.025, respectively). In severe HACE, seven cases showed bilateral corticospinal tract edema at the thalamic level, and SWI showed cerebral microbleeds (CMBs) in five cases, especially in the corpus callosum.

**Conclusions:**

MRI has more advantages than CT in the evaluation of HACE, especially in the DWI sequence. The white matter injury of severe HACE is more severe and extensive, especially in the corpus callosum, and some CMBs and corticospinal tract edema may also appear.

## Introduction

High-altitude cerebral edema (HACE) is a serious and potentially fatal disease that often occurs at high altitudes and is usually considered the most severe form of mountain sickness (AMS) (1–3). In China, HACE is also classified as a severe acute high-altitude disease (AHAD) (4). HACE is common among people who have lived at low altitudes for an extended period and suddenly ascend to altitudes above 3,000 m, especially if the ascent is rapid. The high-altitude environment, which is characterized by low pressure, hypoxia, cold temperature, and dryness, can cause a range of physiological changes in the body. If the body is unable to adapt, HACE may occur (5, 6). HACE is usually characterized by ataxia, fatigue, and changes in mental state. Other clinical manifestations include headaches, behavioral changes, hallucinations, disorientation, decreased levels of consciousness, focal neurological signs, and coma, which are also consistent with the manifestations of organic brain syndrome (3). If not diagnosed and treated in time, the patient may rapidly progress to a coma and die within 24 h (7–9). Previous studies mostly focused on CT scans (10, 11) or conventional MRI sequences (T1WI, T2WI, and FALIR) (10, 12, 13) scans, with only a few studies using functional imaging techniques such as DWI and SWI (14). In this study, we performed not only cranial CT and multi-sequence MRI (T1WI, T2WI, FLAIR, and DWI) scans on both groups but also SWI scans on some patients with HACE. To the best of our knowledge, this is the first study to measure the ADC value of the splenium of the corpus callosum in patients with HACE, which can help objectively and quantitatively evaluate the severity of HACE injury. Therefore, our study offers a more comprehensive examination of the imaging features of HACE and provides valuable guidance for the early diagnosis and treatment of patients with HACE.

## Materials and methods

### Clinical data

This study was approved by the ethics committee of our hospital (approval number: P-SL-2022-014). In this study, we selected 30 patients diagnosed with HACE at our hospital from January 2012 to August 2022 as the experimental group, including six women and 24 men with an average age of 37.40 ± 11.72 years. Based on the clinical symptoms, patients with HACE were divided into mild (characterized by symptoms such as headache, dizziness, nausea, vomiting, limb weakness, and other symptoms) and severe (characterized by symptoms such as limb convulsions, disturbance of consciousness, and other neuropsychiatric symptoms). Among the 30 patients with HACE, 17 patients ascended from the plain to a plateau above 3,000 m, while 13 patients ascended from an altitude of around 2,000 m to an altitude above 3,500 m. All patients completed the relevant examination within 15 days. The inclusion criteria were as follows: (1) meeting the diagnostic criteria for HACE as outlined by the third National Symposium on High Altitude Medicine of the Chinese Medical Association (4) and (2) no contraindications to CT or MRI. The exclusion criteria were as follows: (1) acute cerebrovascular disease, acute drug or carbon monoxide poisoning, epilepsy, meningitis, and encephalitis; (2) any current or past organic diseases. In addition, we selected 60 patients with dizziness who were short time from the plain to a plateau of above 3,000 m or from an altitude of around 2,000 m to an altitude above 3,500 m in the same time period as the control group. The control group had a similar male-to-female ratio and the same average age as the experimental group to eliminate baseline differences. The inclusion criteria were as follows: (1) normal head CT and MRI and (2) no contraindications to CT or MRI. The exclusion criterion was as follows: (1) any current or past organic diseases.

### Instruments and methods

#### MRI examination method

This study obtained MRI data from Siemens Prisma 3.0T and Philips Achieva 3.0T magnetic resonance scanners using a head coil. A total of 90 patients were scanned in the supine position, with the TIWI, T2WI, FLAIR, and DWI sequences obtained in the transverse position and the SWI sequence obtained in nine cases of the transverse position. The following scanning parameters were used: T1WI sequence slice thickness is 5 mm, distance factor is 30%, slices are 21, voxel size is 0.60 × 0.60 × 5.00 mm, and TR/TE/FOV/MAT is 150 ms/2.5 ms/230 mm/256 × 256. T2WI sequence slice thickness is 5 mm, distance factor is 30%, slices are 21, voxel size is 0.72x0.72x5.00mm, and TR/TE/FOV/MAT is 5,000 ms/117 ms/230 mm/256 × 256. FLAIR sequence slice thickness is 5 mm. Distance factor is 30%, slices are 21, voxel size is 0.72 × 0.72 × 5.00 mm, and TR/TE/FOV/MAT is 8,000 ms/81 ms/230 mm/256 × 256. DWI sequence slice thickness is 5 mm, distance factor is 30%, slices are 21, voxel size is 1.44 × 1.44 × 5.00 mm, and TR/TE/FOV/MAT is 3,230 ms/65 ms/230 mm/256 × 256. SWI sequence slice thickness is 1.5mm, distance factor is 20%, slices are 80, voxel size is 0.90 × 0.90 × 1.50 mm, and TR/TE/FOV/MAT is 27 ms/20 ms/230 mm/246 × 256.

#### CT examination method

All 90 patients underwent a head CT using a 64-slice spiral CT scanner (Revolution HD; GE Medical Systems) in the supine position. The following scanning parameters were used: tube voltage 120 kV, tube current 10 mA, slice thickness 5 mm, and rotation time 1s/week.

### Diagnostic criteria and definitions

The gold standard for the diagnosis of HACE is based on the diagnostic criteria proposed by the third National Symposium on High Altitude Medicine of the Chinese Medical Association (4). The direct manifestation of mild and severe HACE on CT scans was a bilateral white matter density decrease, especially in the corpus callosum. As the disease progressed, the decrease in white matter density gradually became more pronounced, and indirect manifestations included the narrowing or even disappearance of the ventricles, the cisterns, and the sulci. The direct manifestations of mild and severe HACE on MRI were hypointensity on T1WI, hyperintensity on T2WI, hyperintensity on FLAIR, hyperintensity on DWI, and a decreased ADC value; indirect manifestations included the narrowing or even disappearance of the ventricles, the cisterns, and the sulci.

In the T1WI sequence, when the signal intensity is slightly lower than the normal white matter, it is called slight hypointensity. When the signal intensity is significantly lower than the normal white matter, it is called obvious hypointensity. In T2WI and FLAIR sequences, when the signal intensity is slightly higher than normal white matter, it is called slight hyperintensity. When the signal intensity is significantly higher than the normal white matter, it is called obvious hyperintensity (15). In the DWI sequence, when the signal intensity is slightly higher than the normal white matter, it is called slight hyperintensity. When the signal intensity is significantly higher than the normal white matter, it is called obvious hyperintensity. We divided the white matter into the juxtaventricular white matter, the periventricular white matter, the deep white matter, and the juxtacortical white matter (16).

### Image analysis

Two radiologists with over 10 years of work experience independently evaluated the head CT images of the two groups of patients without knowing any patient information. In cases of discrepancies, the radiologists discussed and resolved them until a consensus was reached. The incidence of HACE was then determined based on this evaluation.

We named the TIWI, T2WI, and FLAIR sequences conventional MRI sequences. Two other radiologists with more than 10 years of work experience analyzed the two groups' conventional MRI sequences, DWI sequences, and SWI sequences without knowing any patient case data. When there were differences in the results, the two radiologists further discussed and resolved them until a consensus was reached. Then, the incidence of HACE and CMBs and the distribution characteristics of HACE were calculated. Finally, the types were further classified based on the distribution characteristics of edema in the corpus callosum region.

To quantify the ADC value of the splenium of the corpus callosum, we imported the DWI sequence images into the post-processing software (Philips IntelliSpace Portal). The ADC value of the edema area was then measured three times using the MR Diffusion program, and the ADC value (ADCmean) was calculated. The region of interest (ROI) was placed in the most obvious area of the corpus callosum edema and was slightly smaller than the edema range to minimize the partial volume effect.

### Statistical methods

The data were analyzed using SPSS 25.0 statistical software. The intraclass correlation coefficient (ICC) was used to evaluate the consistency of image analysis and parameter measurement among the four radiologists. An ICC value >0.75 indicates good consistency. The data were expressed as numbers (n) or percentages (%). The normal distribution of count data was analyzed using the two-sample *t*-test for comparison, while the non-normal distribution of count data were analyzed using the Mann–Whitney *U* test for comparison. Qualitative two-category data were compared using the Chi-square or McNemar test, and qualitative two-category grade data were compared using the Wilcoxon signed-rank test. A *p* < 0.05 was considered statistically significant.

## Result

### Demographic information

According to clinical symptoms, 30 cases of HACE were divided into 12 mild cases and 18 severe cases. The levels of leukocytes, neutrophils, and glucose increased to varying degrees in both mild and severe HACE cases. There was no significant difference in general clinical data between the two groups (all *p* > 0.05), as shown in Table 1.

**Table 1:** Clinical data of 30 patients with mild and severe HACE.

**Classification**	**Mild HACE**	**Severe HACE**	**95% confidence interval for difference**	***p***
Age (years)	33.83 ± 12.24	39.78 ± 11.06	−5.94	0.178
Mean±SD			(–14.75–2.87)	
Sex (N, %)				0.660
Men	9(75%)	15(83%)		
Women	3(25%)	3(17%)		
Leukocytes	12.77	12.21	0.66	0.767
M (P_25_, P_75_)	(9.94,18.32)	(10.01,15.58)	(–2.81–4.66)	
Neutrophil	81.20	85.96	−2.43	0.397
M (P_25_, P_75_)	(78.06,85.26)	(77.31,91.05)	(–8.30–4.17)	
Glucose content	6.70	7.09	−0.54	0.459
M (P_25_, P_75_)	(5.68,7.80)	(6.09,9.26)	(–2.12–0.90)	

### Consistency of image analysis and ADC value measurement

The consistency test was used to evaluate the ICC of the two radiologists who analyzed CT images for HACE; their ICC was 0.85. The other two radiologists evaluated MRI images for the presence of HACE and white matter edema in the juxtaventricular, periventricular, deep, and juxtacortical areas, as well as edema in the corpus callosum and the corticospinal tract, and the presence of CMBs and signal intensity in each sequence. The ICC for these observations was 1.00. The ICC of the ADC values measured by the two radiologists was 0.98.

### Head CT findings

**Figure 1:**
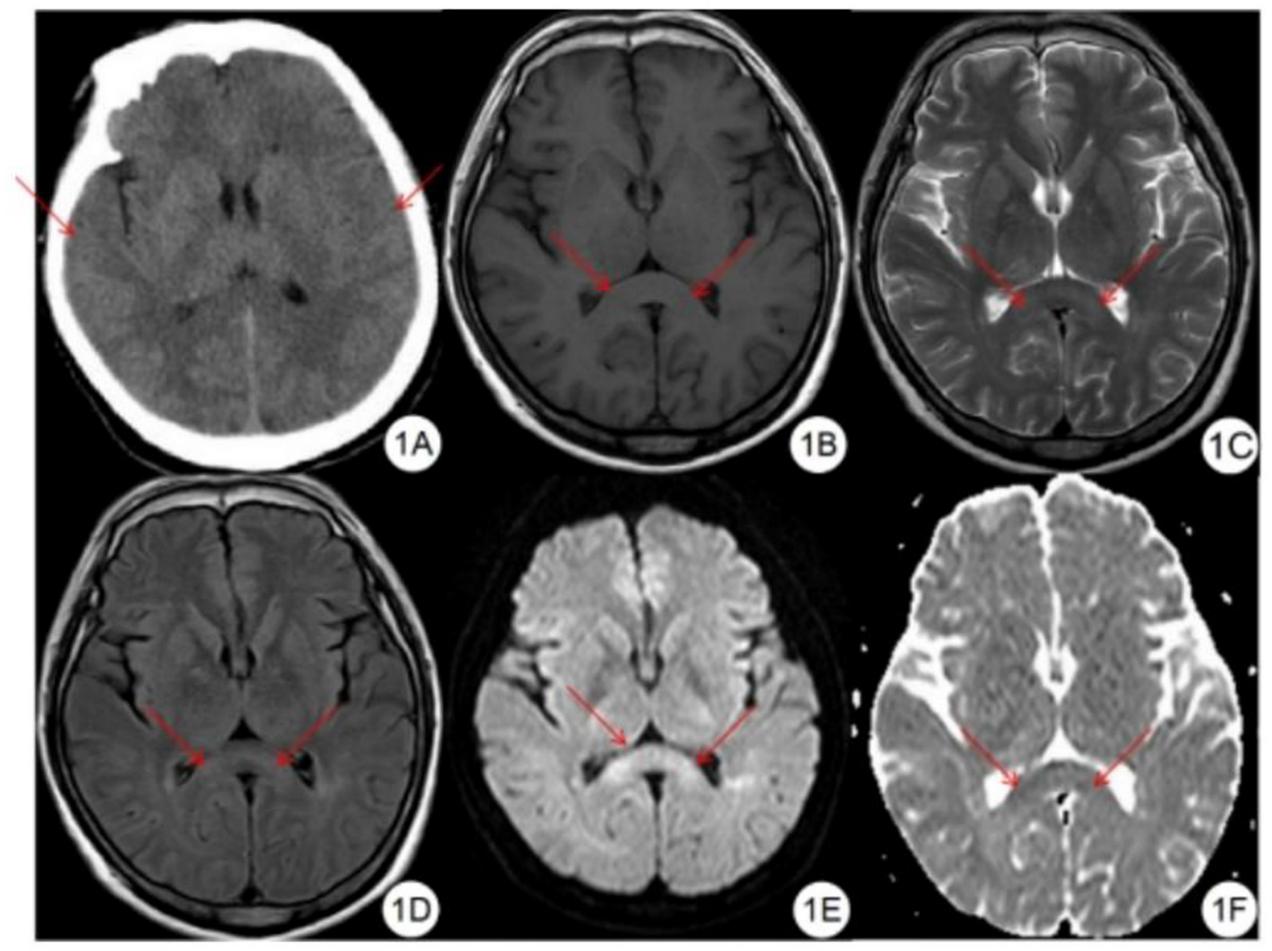
Mild HACE patient. **(A)** CT plain scan showed that the sulcus and cerebral fissure were slightly shallower. **(B)** T1WI showed symmetrical slightly hypointensity in the splenium of the corpus callosum. **(C)** T2WI showed symmetrical slightly hyperintensity in the splenium of the corpus callosum. **(D)** FLAIR showed symmetrical slightly hyperintensity in the splenium of the corpus callosum. **(E)** DWI showed symmetrical hyperintensity in the splenium of the corpus callosum. **(F)** ADC shows symmetrical hypointensity in the splenium of the corpus callosum.

**Figure 2:**
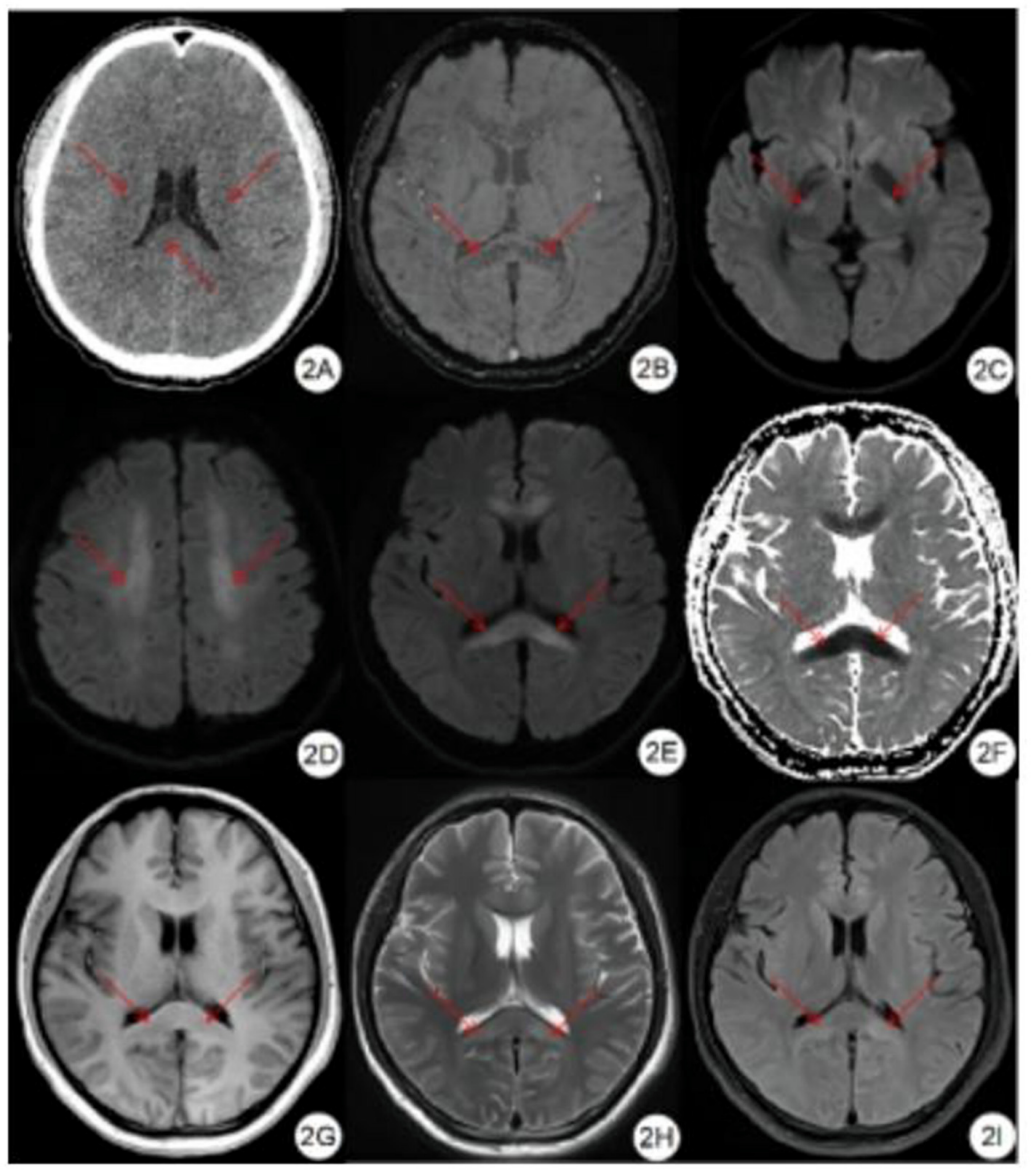
Severe HACE patient. **(A)** CT plain scan showed decreased density of bilateral corona radiata and corpus callosum, and shallow sulci and fission. **(B)** SWI showed symmetrical diffuse speckled hypointensity in the brain parenchyma, especially in the splenium and genu of the corpus callosum. **(C–E)** DWI showed symmetrical hyperintensity in bilateral corticospinal tract, centrum semiovale, splenium and genu of the corpus callosum. **(F)** ADC showed symmetrical hypointensity in the splenium and genu of the corpus callosum. **(G)** T1WI showed symmetrical hypointensity in the splenium and genu of the corpus callosum. **(H)** T2WI showed symmetrical hyperintensity in the splenium and genu of the corpus callosum. **(I)** FLAIR showed symmetrical hyperintensity in the splenium and genu of the corpus callosum.

Compared with the gold standard, the sensitivity of CT diagnosis was 23.3% and the specificity was 100%, as shown in Table 2. Of the 12 patients with mild HACE, 1 (8.3%) had brain swelling, and none (0%) had corpus callosum edema, as shown in Figure 1. Among the 18 patients with severe HACE, 6 (33.3%) had brain swelling, and 2 (11.1%) had corpus callosum edema, as shown in Figure 2.

**Table 2:** Positive distribution of CT and gold standard examination in 90 cases.

**CT**	**Gold standard**		**Total**
	**Positive**	**Negative**	
Positive	7	0	7
Negative	23	60	83
Total	30	60	90

### Head MRI findings

**Figure 3:**
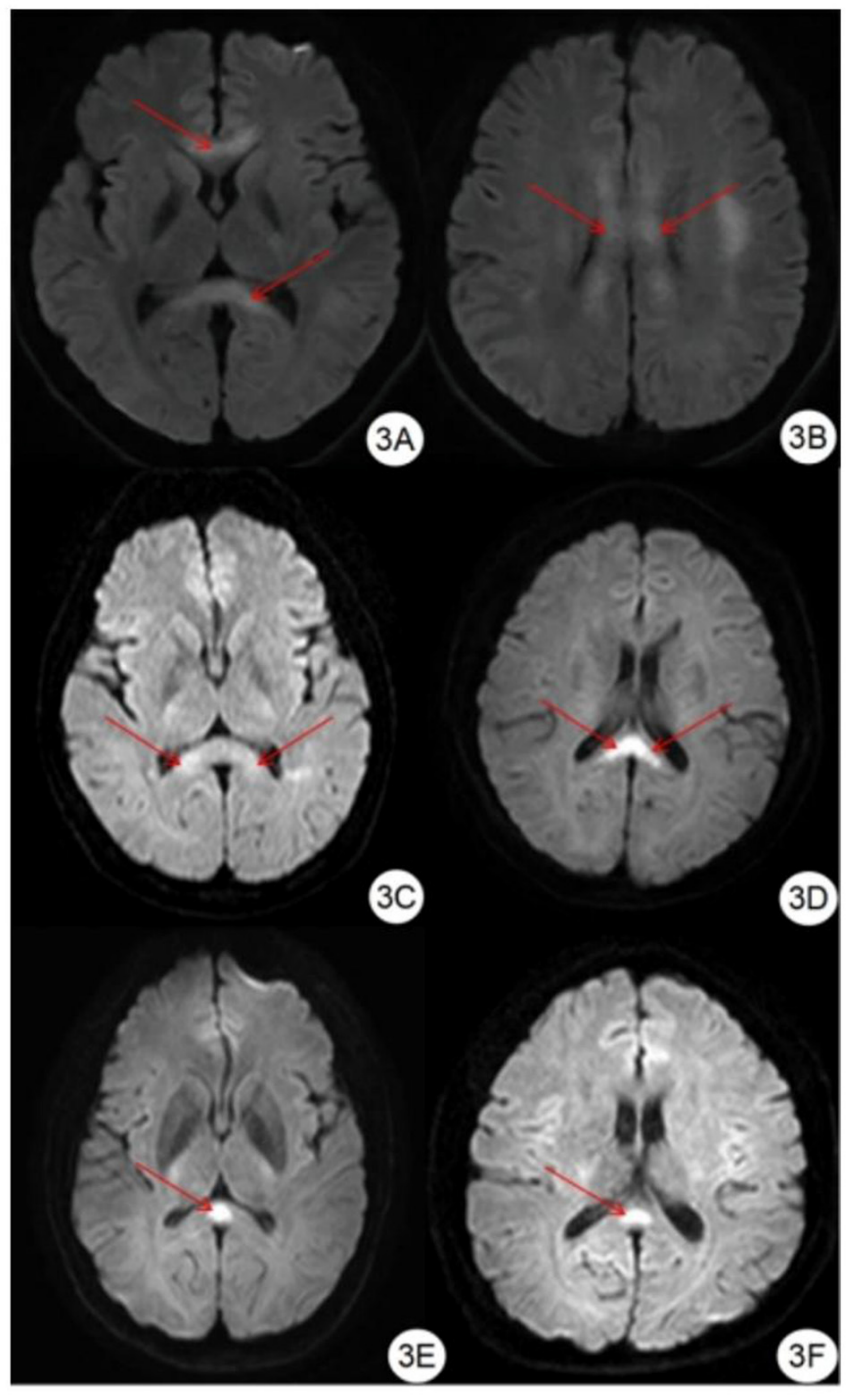
Classification of edema in corpus callosum region. **(A, B)** DWI showed symmetrical hyperintensity in the splenium, trunk, and genu of the corpus callosum. **(C, D)** DWI showed symmetrical hyperintensity in the splenium of the corpus callosum. **(E, F)** DWI showed hyperintensity in the center of the splenium of the corpus callosum.

Compared with the gold standard, the sensitivity and specificity of MRI were both 100%, as shown in Table 3. All patients with HACE had intracranial symmetrical white matter edema, particularly in the splenium of the corpus callosum. Seven patients with severe HACE (38.9%) had significant edema of the corticospinal tract at the bilateral thalamic level, as shown in Figure 2. Table 4 shows that the range of deep and juxtacortical white matter edema in severe HACE was significantly larger than in patients with mild HACE (*p* < 0.001). Figure 1 shows that all 12 patients with mild HACE had juxtaventricular and periventricular white matter edema, while none had deep and juxtacortical white matter edema. In contrast, Figure 2 shows that all 18 patients with severe HACE had juxtaventricular and periventricular white matter edema, with 13 (72.2%) also having deep and juxtacortical white matter edema. In mild HACE, the signal intensity of DWI sequence was significantly higher than that of conventional MRI sequence (*p* = 0.008), as shown in Table 5. In severe HACE, the signal intensity of DWI sequence was significantly higher than that of conventional MRI sequences (*p* = 0.025), as shown in Table 6. Table 7 shows that the range of corpus callosum edema in severe HACE was significantly larger than in patients with mild HACE (*p* = 0.001). In the 12 patients with mild HACE, 10 (83.3%) had localized central edema in the splenium of the corpus callosum and 2 (16.7%) had localized symmetrical edema. In the 18 patients with severe HACE, 3 (16.7%) had localized central edema, 8 (44.4%) had localized symmetrical edema, and 7 (38.9%) had diffuse symmetrical edema in the splenium of the corpus callosum, as shown in Figure 3. The SWI sequence showed that five of the nine patients (55.6%) with severe HACE had CMBs, particularly in the corpus callosum. One patient with diffuse CMBs died, as shown in Figure 2. The edema in the white matter of patients with mild and severe HACE was caused by ischemia and hypoxia, and DWI showed hyperintensity. The ADC values decreased to varying degrees, with the ADC value of the splenium of the corpus callosum in severe HACE being significantly lower than that in mild HACE (*p* = 0.049), as shown in Table 8.

**Table 3:** Positive distribution of MRI and gold standard examination in 90 cases.

**MRI**	**Gold standard**		**Total**
	**Positive**	**Negative**	
Positive	30	0	30
Negative	0	60	60
Total	30	60	90

**Table 4:** Distribution of deep and juxtacortical white matter edema in 30 HACE.

**Grouping**	**Total (case)**	**Deep and juxtacortical white matter edema positive [case (%)]**	**Deep and juxtacortical white matter edema negative [case (%)]**	***Fisher's Exact Test***
				***p***
Mild HACE	12	0(0%)	12(100%)	< 0.001
Severe HACE	18	13(72.2%)	5(27.8%)	

**Table 5:** Signal intensity distribution of mild HACE DWI sequence and MRI ordinary sequence in 12 cases.

**DWI**	**MRI ordinary sequence**		**Total**	***p***
	**Signal intensity is obvious**	**Signal intensity is slightly obvious**		
Signal intensity is obvious	0	7	7	0.008
Signal intensity is slightly obvious	0	5	5	
Total	0	12	12	

**Table 6:** Signal intensity distribution of severe HACE DWI sequence and MRI ordinary sequence in 18 cases.

**DWI**	**MRI ordinary sequence**		**Total**	***p***
	**Signal intensity is obvious**	**Signal intensity is slightly obvious**		
Signal intensity is obvious	7	8	15	0.025
Signal intensity is slightly obvious	0	3	3	
Total	7	11	18	

**Table 7:** Distribution of edema in the corpus callosum in 30 cases of HACE.

**Grouping**	**Total (case)**	**Localized central edema**	**Localized symmetrical edema**	**Diffuse symmetrical edema**	***Fisher Exact Test***
		**[case(%)]**	**[case(%)]**	**[case(%)]**	***p***
Mild HACE	12	10(83.3%)	2(16.7%)	0(0%)	0.001
Severe HACE	18	3(16.7%)	8(44.4%)	7(38.9%)	

The classification definitions for edema in the corpus callosum region are as follows:

Diffuse symmetrical edema of the corpus callosum: edema involves more than two areas in the splenium, trunk, and genu of the corpus callosum.

Localized edema of the splenium of the corpus callosum: edema only involves the splenium of the corpus callosum.

When the area of edema symmetrically involves only both sides of the splenium of the corpus callosum (including or excluding the central area), it is called localized symmetrical edema of the splenium of the corpus callosum.

When the edema only involves the central splenium of the corpus callosum, it is called localized central edema of the splenium of the corpus callosum.

**Table 8:** The ADC mean values of paraventricular and periventricular (splenium of corpus callosum) white matter edema in 30 patients with mild and severe HACE (X10^−3^mm^2^/s).

**Grouping**	**Mild HACE**	**Severe HACE**	***P***
ADC mean value of splint of corpus callosum M(P25, P75)	0.63 (0.59,0.68)	0.52 (0.33,0.62)	0.049

## Discussion

### Evaluation and analysis of HACE using CT

On a CT scan, HACE may be directly indicated by decreased bilateral white matter density and indirectly by narrowed or even disappearing ventricles and sulci. In mild or early-stage HACE, the decrease in the CT values of the white matter may not be visible due to the mild degree of edema. The poor resolution of gray and white matter on a CT scan can make it difficult for radiologists, particularly those with less diagnostic experience, to detect HACE. This may result in a misdiagnosis of HACE. The study by Li et al. (10) found that, among the 36 patients diagnosed with HACE, only one patient had abnormal findings on the head CT. In the study by Shen et al. (11), only 11 of 32 patients with HACE had abnormal findings on CT. In our study, only 7 of 30 patients with HACE had abnormal findings on CT, including one patient with mild HACE and six patients with severe HACE. The sensitivity of CT diagnosis was 23.3%, whereas the sensitivity of MRI for HACE diagnosis was 100%. Therefore, we believe that head CT is less sensitive to HACE than MRI and that it is easy to miss a diagnosis, especially for mild HACE. In multimodal MRI scanning, not only the range of HACE damage can be clearly displayed but also the ADC value can be measured specifically to evaluate the damage degree of HACE more objectively and quantitatively. In addition, through multimodal MRI, we can also observe other complications caused by HACE, such as CMBs, to provide more useful information for diagnosing and treating patients. Therefore, when HACE is suspected, a head MRI, not only a head CT, should be performed in time, especially when HACE is mild or the symptoms are mild.

### Pathophysiological and corresponding MRI findings of HACE

Currently, the pathophysiological mechanism of HACE is considered to be cytotoxic and vasogenic (3). Vasogenic edema refers to extracellular water accumulation caused by increased blood–brain barrier permeability (17). Hypoxia is caused by the interaction of neurohormones such as vascular endothelial growth factor, nitric oxide, reactive cytokines, and free radicals with hemodynamic responses, which leads to microvascular capillary bed leakage, destroying the blood–brain barrier and resulting in an increase in intracranial pressure (18). Cytotoxic edema refers to the formation of free radicals due to hypoxia, leading to impaired Na+/K+-APT enzymes, oxidative stress, swelling of astrocytes, and the resulting cytotoxic edema (17). When people first arrive from the plain to the plateau, or from the high altitude to the higher altitude in a short time, if they encounter the influence of some external environmental factors, such as chill, cold, decreased immunity, and so on, the tolerance of the body to hypoxia decreases. The brain consumes copious amounts of oxygen, making it highly sensitive to hypoxia (19–21). Hypoxia can lead to swelling of brain cells, which manifests as prolonged relaxation time on T1WI and T2WI, that is, hypointensity on T1WI and hyperintensity on T2WI. In the early stage of HACE, due to the dysfunction of the cellular sodium-potassium pump during hypoxia, the intracellular water molecules increase and swell, which restricts the diffusion of water molecules; that is, the DWI shows hyperintensity, and the ADC value decreases significantly. At this time, the signal performance on T1WI, T2WI, and FLAIR may not be noticeable or even absent (12). The study by Li et al. (10) found that, among the 36 patients diagnosed with HACE, 22 patients had abnormal signals of MRI in the brain parenchyma in the early stage of HACE, that is, before the onset of mental and neurological symptoms, showing speckled and patchy T1WI hypointensity and T2WI hyperintensity. In this study, different degrees of signal abnormalities were found in the areas of white matter edema in 30 patients with HACE on each sequence of MRI. In mild and severe HACE, the signal intensity of the DWI sequence was significantly higher than that of the conventional MRI sequence, and the difference was statistically significant, especially in mild HACE. Therefore, we believe that the DWI sequence has the greatest advantage in the evaluation of white matter damage in HACE, especially in mild HACE.

### Evaluation and analysis of HACE by MRI

On MRI, HACE shows that T1WI hypointensity, T2WI hyperintensity, FLAIR hyperintensity, DWI hyperintensity, and ADC value decreases in varying degrees. The study by Chen et al. (13) found that HACE showed diffuse changes on MRI and that the corpus callosum, cerebrum, thalamus, brainstem, and cerebellum could all be involved, especially in the corpus callosum, with certain characteristics. Tan et al. (12) found that HACE was involved in the corpus callosum, the juxtacortical white matter, the cerebellum, the cingulate gyrus, and the septum pellucida, T1WI showed hypointensity, T2WI showed hyperintensity, FLAIR, and DWI showed hyperintensity. In the study by PH Hackett et al. (14), eight patients with HACE showed T2WI and FALIR hyperintensity in the first MRI scan, mainly involving the corpus callosum, the juxtacortical white matter, and the periventricular white matter and mainly involving the corpus callosum and the juxtacortical white matter, respectively. Some scholars (18, 22) also reported that patients with HACE have hypointensity on T1WI, hyperintensity on T2WI, and FLAIR in the white matter area centered on the corpus callosum, while patients who underwent DWI sequence scanning showed hyperintensity due to restricted diffusion. In this study, symmetrical edema of the juxtaventricular and periventricular white matter occurred in all patients with HACE, and 13 out of 18 patients with severe HACE had deep and juxtacortical white matter edema, which was consistent with previous studies. It was found that both mild and severe HACE showed different degrees of juxtaventricular and periventricular white matter edema, while the distribution of deep and juxtacortical edema in severe HACE was significantly larger than that in mild HACE, indicating that, with the aggravation of HACE, the scope of edema gradually spread from the juxtaventricular and periventricular white matter to the deep and juxtacortical white matter. This study also found that the main manifestations in 30 patients with HACE were symmetrical white matter edema, especially in the splenium of the corpus callosum. We further classified the patients according to corpus callosum edema distribution characteristics. We found that the distribution range of corpus callosum edema in severe HACE was significantly larger than that in mild HACE. The ADC value in the splenium edema area of severe HACE was significantly lower than that in mild HACE, and the above differences were statistically significant. Therefore, we believe that the edema caused by ischemia and hypoxia in severe HACE is more extensive and severe. In addition, edema of the corticospinal tract at the bilateral thalamic plane was found in 7 patients with severe HACE, leading to impaired autonomic motor function such as limb convulsions.

### Evaluation and analysis of HACE by SWI

Cerebral microbleeds (CMBs) are defined as small (< 10 mm), hypointensity, round or oval lesions that can be detected using the T2^*^ gradient-weighted echo and SWI sequence (23–25), and there is no edema around them (26). A histopathological examination showed that CMBs were a punctate hemorrhagic focus containing hemosiderin deposits, probably caused by leakage of red blood cells in small cerebral vessels (such as arterioles and capillaries) (25), caused by blood exosmosis caused by degeneration of small vessels, and the subsequent decomposition of hemoglobin released from red blood cells (27, 28). CMBs are related to cerebral hemorrhage, ischemic stroke, dementia, Parkinson's disease, Alzheimer's disease, etc. (25, 29–33) and can also be seen in the injury caused by high altitude hypoxia (34). In the study by PH Hackett et al. (14), six patients who underwent SWI all showed extensive CMB signs, especially in the corpus callosum and the juxtacortical white matter. Some scholars also reported extensive CMBs in patients with HACE, especially in the corpus callosum, the white matter, and the semiovale center (18, 35, 36). In this study, five out of 9 patients with severe HACE who underwent SWI scans showed CMBs, which were significant in the corpus callosum, and one patient with diffuse CMBs died. Some studies suggest that CMBs can lead to cognitive dysfunction and increase the risk of stroke (28, 33) and even death (37). Therefore, we believe that patients developing CMBs often have poor prognoses and should be treated promptly.

### Deficiency and prospects

Many patients with HACE travel to high-altitude areas for the first time and return to lower altitudes shortly after their symptoms improve. Therefore, we cannot follow-up with some of these patients. This study found that some patients with severe HACE had bilateral corticospinal tract edema. In the future, we will perform DTI to more accurately evaluate the injury to the corticospinal tract in patients with HACE.

## Conclusion

In conclusion, HACE is a condition characterized by ischemic and hypoxic changes in the white matter area, with the splenium of the corpus callosum being the most sensitive region. Severe HACE causes more extensive and severe white matter damage compared to mild HACE. CT scans are less sensitive to mild HACE and may result in misdiagnosis, while MRI is more effective in detecting intracranial edema in HACE, especially when using the DWI sequence. The extent of corpus callosum edema is greater in severe HACE than in mild HACE. Patients with severe HACE can also cause CMBs and bilateral thalamic-level corticospinal tract edema. The presence of CMBs often indicates the deterioration of the disease and may lead to a poor prognosis, which should be promptly treated.

## Data availability statement

The raw data supporting the conclusions of this article will be made available by the authors, without undue reservation.

## Ethics statement

The studies involving human participants were reviewed and approved by Ethics Committee of Qinghai University Affiliated Hospital. Written informed consent from the patients/participants or patients/participants' legal guardian/next of kin was not required to participate in this study in accordance with the national legislation and the institutional requirements.

## Author contributions

HB and CL: conception, design, and supervision. HB: administrative support, funding acquisition, and final approval of manuscript. CL: provision of study materials or patients, collection and assembly of data, data analysis, interpretation, software and validation, visualization, and manuscript writing. All authors contributed to the article and approved the submitted version.
